# Characterization and analysis of the cotton cyclopropane fatty acid synthase family and their contribution to cyclopropane fatty acid synthesis

**DOI:** 10.1186/1471-2229-11-97

**Published:** 2011-05-25

**Authors:** Xiao-Hong Yu, Richa Rawat, John Shanklin

**Affiliations:** 1 Department of Biochemistry and Cell Biology, Stony Brook University, NY, USA; 2 Biology Department, Brookhaven National Laboratory, Upton, NY, USA

## Abstract

**Background:**

Cyclopropane fatty acids (CPA) have been found in certain gymnosperms, Malvales, Litchi and other Sapindales. The presence of their unique strained ring structures confers physical and chemical properties characteristic of unsaturated fatty acids with the oxidative stability displayed by saturated fatty acids making them of considerable industrial interest. While cyclopropenoid fatty acids (CPE) are well-known inhibitors of fatty acid desaturation in animals, CPE can also inhibit the stearoyl-CoA desaturase and interfere with the maturation and reproduction of some insect species suggesting that in addition to their traditional role as storage lipids, CPE can contribute to the protection of plants from herbivory.

**Results:**

Three genes encoding cyclopropane synthase homologues GhCPS1, GhCPS2 and GhCPS3 were identified in cotton. Determination of gene transcript abundance revealed differences among the expression of GhCPS1, 2 and 3 showing high, intermediate and low levels, respectively, of transcripts in roots and stems; whereas GhCPS1 and 2 are both expressed at low levels in seeds. Analyses of fatty acid composition in different tissues indicate that the expression patterns of GhCPS1 and 2 correlate with cyclic fatty acid (CFA) distribution. Deletion of the N-terminal oxidase domain lowered GhCPS's ability to produce cyclopropane fatty acid by approximately 70%. GhCPS1 and 2, but not 3 resulted in the production of cyclopropane fatty acids upon heterologous expression in yeast, tobacco BY2 cell and Arabidopsis seed.

**Conclusions:**

In cotton GhCPS1 and 2 gene expression correlates with the total CFA content in roots, stems and seeds. That GhCPS1 and 2 are expressed at a similar level in seed suggests both of them can be considered potential targets for gene silencing to reduce undesirable seed CPE accumulation. Because GhCPS1 is more active in yeast than the published *Sterculia *CPS and shows similar activity when expressed in model plant systems, it represents a strong candidate gene for CFA accumulation via heterologous expression in production plants.

## Background

Fatty acids containing three-carbon carbocyclic rings, especially cyclopropane fatty acids, occur infrequently in plants and their major plant producers include Malvaceae, Sterculiaceae, Bombaceae, Tilaceae, Gnetaceae and Sapindaceae [1-4]. They can represent a significant component of seed oils and accumulate to as much as 40% in *Litchi chinensis *[1,5].

Cyclopropane synthases (CPSs) catalyze the cyclopropanation of unsaturated lipids in bacteria [6,7], plants [8,9] and parasites [10]. There are two principle classes of bacterial cyclopropane synthases: the *Escherichia coli *cyclopropane synthase (ECPS) type that uses unsaturated phospholipids as substrates and *Mycobacterium tuberculosis *cyclopropane mycolic acid synthases (CMAs) that perform the introduction of *cis*-cyclopropane rings at proximal and distal positions of unsaturated mycolic acids [11-14]. Despite their different substrates the two classes of enzymes share up to 33% sequence identity suggesting a common fold and reaction mechanism. Moreover, a shared reaction mechanism is suggested by the fact that both *E. coli *CPS and *M. tuberculosis *CMA active site residues are almost completely conserved and harbor a bicarbonate ion in their active site [15,16].

Although CPA had been identified in a few plant seeds as early as 1960s [17], the key gene responsible for their biochemical synthesis was not identified for more than three decades when Bao et al. [8] identified a cyclopropane synthase from *S. foetida*. The *Sf*CPS is a microsomal-localized membrane enzyme, which catalyzes the addition of a methylene group derived from *S*-adenosyl-L-methionine across the double bond of oleic acid esterified to the *sn*-1 position of PC [9]. The *S. foetida *enzyme is the first plant CPS that has been characterized, the other plant CPS has been reported to date is from *Litchi sinensis *(WO/2006087364).

*E. coli *CPS is thought to be involved in the long-term survival of non-growing cells and its expression can be associated with environmental stresses [6]. Plant CPEs inhibit some insect stearoyl-CoA desaturases thereby interfering with their maturation and reproduction, suggesting that in addition to their role as storage lipids, CPE can also serve as protective agents. CPE are also strong inhibitors of a variety of fatty acid desaturases in animals [18-21], and feeding animals with CPE -containing oilseeds, such as cotton seed meal, leads to accumulation of hard fats and other physiological disorders [20,22,23]. For the same reasons vegetable oils that contain CPE must be treated with high temperature hydrogenation before human consumption. These treatments add to processing costs and also result in the accumulation of undesirable trans-fatty acids. Therefore, reducing the levels of CPE in cotton seed oil by gene-silencing or other techniques could reduce processing costs and the associated production of undesirable trans fatty acids as well as increasing the value of processed seed meal for food consumption (US2010/0115669).

Cyclic fatty acids, especially CPA such as dihydrosterculic acid, are desirable for numerous industrial applications and therefore it would be useful to identify candidate enzymes for heterologous expression in production plants with the goal of optimizing the accumulation of CPAs. CPAs have physical characteristics somewhere in between saturated and mono unsaturated fatty acids. The strained bond angles of the carbocyclic ring are responsible for their unique chemistry and physical properties. Hydrogenation results in ring opening to produce methyl-branched fatty acids. These branched fatty acids have the low temperature properties of unsaturated fatty acids, but unlike unsaturated fatty acids, their esters are not susceptible to oxidation and are therefore ideally suited for use in lubricant formulations [24] (WO 99/18217). Moreover, the methyl branched fatty acids are an alternative to isostearic acids that are used as cosmetics. Oils with high levels of cyclopropene fatty acids self-polymerize at elevated temperatures because the cyclopropene ring is highly strained and readily opens in an exothermic reaction. This property makes CPE particularly suitable for the productions of coatings and polymers. Sterculic acid (18-carbon cyclopropene) also has potential applications as a biocide in fatty acid soap formulation (US2008/0155714A1).

In this study, we identify three CPS isoforms from cotton and analyze their expression in different tissues to help define their physiological roles. We also present an analysis of the consequences of over-expressing cotton *GhCPS1, 2 and 3 *in yeast, tobacco suspension cells and Arabidopsis.

## Methods

### Plant growth conditions and transgenic analyses

Arabidopsis and camelina plants were grown in walk-in-growth chambers at 22°C for 16 h photoperiod, and cotton plants were grown in the greenhouse at 28°C for 16 h photoperiod. The full length cDNA corresponding to *GhCPS1*[GenBank:574036.1]*, GhCPS2 *[GenBank:574037.1] and *GhCPS3*[GenBank:574038.1] genes were PCR amplified using gene specific primers with restriction site-encoding linkers and subsequently digested and cloned into pYES2 (Invitrogen) via corresponding SacI and EcoRI restriction sites. For Arabidopsis and camelina transformation, the genes were cloned downstream of the phaseolin seed-specific promoter in binary vector pDsRed [25]. These binary vectors were introduced into *Agrobacterium tumefaciens *strain GV3101 by electroporation and were used to transform Arabidopsis via the floral dip method [26], and camelina through vacuum infiltration [27]. Seeds of transformed plants were screened under fluorescence, emitted upon illumination with green light from a X5 LED flashlight (Inova) in conjunction with a 25A red camera filter [25]. For tobacco Bright Yellow 2 (BY2) transformation, GhCPS1 was cloned into pBI121 using BamHI and SacI sites, and GhCPS2 and 3 were cloned into pBI121 using the XbaI and SacI restriction sites and transformed into BY2 cells. After 4-5 months kanamycin selection, stable transformed cell lines were collected 7 days after subculture and analyzed for fatty acid composition. The composition of pBI121-containing negative control lines were compared with lines transformed with SfCPS in pBI121.

Primers used in this study for:

Yeast expression

GCPS1-Y2F: ACCGGAGCTCAcca t g g a a g t g g c c g t g a t c g

GCPS2-Y2F: ACCGGAGCTC Acca t g g a a g t g g c g g t g a t c g

GCPS1+2-R: CCGGAATTC t c a a t c a t c c a t g a a g g a a t a t g c

GCPS3-Y2F: ACCGGAGCTC AccATGGGTa t g a a a a t a g c a g t g a t a g g a g g a g

GCPS3-R: CCGGAATTC t t a a g a a g c t g a g g g g a a g t c t t t

Arabidopsis transformation

GCPS1-5'PacI: tcccTTAATTAA a t g g a a g t g g c c g t g a t c g

GCPS2-5'PacI: tcccTTAATTAA a t g g a a g t g g c g g t g a t c g

GCPS1+2-3'XmaI: tcccCCCGGG t c a a t c a t c c a t g a a g g a a t a t g

SfCPS-5'PacI: tcccTTAATTAA a t g g g a g t g g c t g t g a t c g

SfCPS-3'XmaI: tcccCCCGGG t c a a t t a t c c g a g t a g g a a t a t g c

GCPS3-5'PacI: tcccTTAATTAA a t g a a a a t a g c a g t g a t a g g a g g a

GCPS3-3'XbaI: GCTCTAGA t t a a g a a g c t g a g g g g a a g t c t t t

Tobacco BY2 transformation

GCPS3-5'XbaI: GCTCTAGA a t g a a a a t a g c a g t g a t a g g a g g a

GCPS3-3'SacI: ACCGGAGCTC t t a a g a a g c t g a g g g g a a g t c t t t

GCPS2-5'XbaI: GCTCTAGA a t g g a a g t g g c g g t g a t c g

GCPS1+2-3'SacI: ACCGGAGCTC t c a a t c a t c c a t g a a g g a a t a t g

GCPS1-5'BamHI: CGCGGATCC a t g g a a g t g g c c g t g a t c g

SfCPS-5'XbaI: GCTCTAGA a t g g g a g t g g c t g t g a t c g

SfCPS-3'SacI: ACCGGAGCTC t c a a t t a t c c g a g t a g g a a t a t g c

Sequence homologous to the CPS sequences in lower case, flanking sequences in boldface, restriction site sequence underlined.

### Phylogenetic and sequence analysis

Phylogenetic analysis of the cyclopropane fatty acid synthase (CPS) family was conducted by using full length protein sequences from cotton and cyclopropane fatty acid synthase from *Sterculia, E. coli, Agrobacterium, Mycobacterium*, and Arabidopsis. Full-length amino-acid sequences were first aligned by CLUSTALW version 2.0.12 (Additional file 1) [28] with default parameters (http://www.ebi.ac.uk/Tools/clustalw/), and imported into the Molecular Evolutionary Genetics Analysis (MEGA) package version 5.0 [29]. Phylogenetic and molecular evolutionary analyses were conducted using the neighbor-joining (NJ) method [30] implemented in MEGA, with the pair-wise deletion option for handling alignment gaps, and the Poisson correction model for computing distance. The final tree graphic was generated using TreeView program [31].

### RNA extraction, Reverse transcription and quantitative PCR analyses

RNA from cotton leaf, flower, stem and seeds at different development stages were extracted according to Wu et al. [32] and RNA from cotton root was isolated using Qiagen's plant Mini RNA kit. RNA quality and concentration were determined by gel electrophoresis and Nanodrop spectroscopy. Reverse transcription (RT) was carried out using Qiagen's QuanTect Reverse Transcription Kit. Quantitative PCR analyses were carried out using Bio-RAD iQ™ SYBR Green Supermix as described in Additional file [2]. Primers ubiq7-1F (5'- GAAGGCATTCCACCTGACCAAC -3') and Ubiq7-1R (5'- CTTGACCTTCTTCTTCTTGTGCTTG -3') were used to amplify ubiquitin 7 as internal standard. Gene-specific primers used for qRT-PCR analysis were qGCPS-1F(5'- TTAAGTGGTCAACCGGCCATGCAA -3') and qGCPS-1R (5'-TTCTTTGGACTGGGCGGAACAGAA -3'), qGCPS2-1F (5'-ATATTCCCTGGAGGAACC CTG CTT-3') and qGCPS2-1R (5'-AAACCGGCAGCGCAGTAATCGAAA-3') for GhCPS2, and qGCPS3-1F (5'-ACTGGTTGCGAGGTGCATTCTGTT-3) and qGCPS3-1R (5'-TTGGAAAGCGCCAAGCACTGTTGA -3') for GhCPS3.

### Fatty acid analyses

Yeast culture, expression induction, and fatty acid analyses were carried out as described [33]. Lipids were extracted in methanol/chloroform (2:1) from 0.1 g of fresh weight cotton tissue and internal standard heptadecanoic acid was added. The isolated lipid was methylated in 1% sodium methoxide at 50°C for 1 hr and extracted with hexane. To analyze the fatty acids of single seeds, fatty acid methyl esters (FAMEs) were prepared by incubating the seeds with 35 μL 0.2M trimethylsulfonium hydroxide in methanol [34]. For analysis of CFA in BY2 cell lines, FA were extracted from ~0.1 g of BY2 callus tissue, FAMEs were prepared as described above for cotton tissue, or FA dimethyloxazoline (DMOX) derivatives were prepared in a one-pot reaction in which FA are reacted with 2-amino-2-methyl-1-propanol in a nitrogen atmosphere at 190°C for 4 hours [35]. Lipid profiles and acyl group identification were analyzed on a Hewlett Packard 6890 gas chromatograph equipped with a 5973 mass selective detector (GC/MS) and either Agilent J&W DB 23 capillary column (30 m × 0.25 μm × 0.25 μm) or SUPELCO SP-2340 (60 m × 0.25 μm × 0.20 μm) column. The injector was held at 225°C and the oven temperature was varied from 100-160°C at 25°C/min, then 10°C/min to 240°C. The percentage values were converted to mole percent and presented as means of at least three replicates.

## Results

### The cotton cyclopropane fatty acid synthase family consists of three highly conserved members

**Figure 1:**
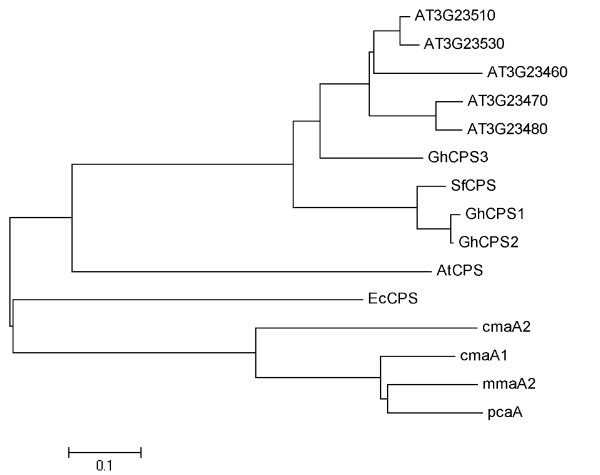
**Phylogenetic tree of cyclopropane synthase genes revealing evolutionary sequence relationships**. The tree was constructed by neighbor-joining distance analysis. Line lengths indicate the relative distances between nodes. Sequences of characterized enzymes and CPS homologues from Arabidopsis and cotton were used for alignment, *pcaA*, mycolic acid synthase from *Mycobacterium tuberculosis *H37Rv [NCBI Reference Sequence: NC_000962.2]; cmaA1, Cyclopropane mycolic acid synthase 1 from *Mycobacterium bovis AF2122/97 *[NCBI Reference Sequence: NC_002945.3]; *AtCPs*, cyclopropane synthase from *Agrobacterium tumefaciens *(AGR-C-3601p); *EcCPS*, cyclopropane synthase from *E.Coli *[NCBI Reference Sequence: NC_000913]; *cmaA2*, Cyclopropane mycolic acid synthase 2 from *Mycobacterium tuberculosis *[NCBI Reference Sequence: NP_215017.1]; *mmaA2*, Cyclopropane mycolic acid synthase MmaA2 from *Mycobacterium tuberculosis *[NCBI Reference Sequence: NP_215158.1].

A database search of the cotton genome database (http://cottondb.org/) identified three genes predicted to encode proteins with high sequence similarity to *Sterculia *CPS (Figure 1). The predicted polypeptides encoded by the cotton CPS isoforms range from 865 to 873 amino acids. Sequence comparisons and phylogenetic analysis of the different isozymes conducted using the MEGA5 program revealed that the GhCPS1 and 2 isozymes are the most similar, showing 97% identity at the amino acid level, and group in a clade with *Sterculia *CPS. GhCPS1 and 2 showed 82% and 84% identity to *Sterculia *CPS, respectively. The GhCPS3 protein showed divergence from these 3 synthases, arising from a completely separate branch (Figure 1). Sequence comparisons showed GhCPS3 had 64% identity with GhCPS1 and 65% with GhCPS2.

The cotton CPSs have two enzymatic domains as reported for the *Sterculia *CPS [8]; the carboxy terminus encodes the CPS domain and catalyzes the synthesis of dihydrosterculate while the amino terminus encodes a distinct oxidase domain of unknown function. A sequence proposed to play a role in S-AdoMet binding, VL(E/D)xGxGxG [36,37], was found in GhCPS3 as ILEIGCGWG and in a more degenerate form (DxGxGxG) in GhCPS1 and 2. Given that S-AdoMet binding and methyl group transfer is the only known function shared between CPS and other S-AdoMet binding enzymes, this segment of the GhCPSs seems very likely to be involved in binding this substrate. It should be noted that all CPS coding sequences lack the phenylalanine residue of the FxGxG, proposed by both Lauster [38] and Smith et al. [39]. However, this motif has been found only in methyltransferases that act on nucleic acids [37].

### Active site conservation

The crystallized *M. tuberculosis *CPS structure shows a bicarbonate ion hydrogen-bonded to five active-site residues [15], including two interactions via backbone amides. In *E.coli *CPS C139, I268, E239, H266, and Y317 are strictly conserved within all non-plant CPSs [16]. In cotton, the amino acids corresponding to *E. Coli *I268, E239, and Y317 are conserved in all 3 GhCPS genes, i.e. I739, E710, Y794 for GhCPS1; I731, E702, Y786 for GhCPS2 and I736, E707, Y791 for GhCPS3. However, H266 has been substituted for Q in GhCPS (Q737, I729 and Q734 respectively). Interestingly, C139 remains the same in GhCPS3 as C602, but is substituted for S in the other two GhCPS (S602 in GhCPS1 and S595 in GhCPS2). An *E. coli *C139S mutant is less active than the wild-type enzyme (its catalytic efficiency is 31%), but addition of bicarbonate increases its K_cat_, by a factor of two [16].

### The GhCPS genes exhibit tissue-specific differences in their expression

**Figure 2:**
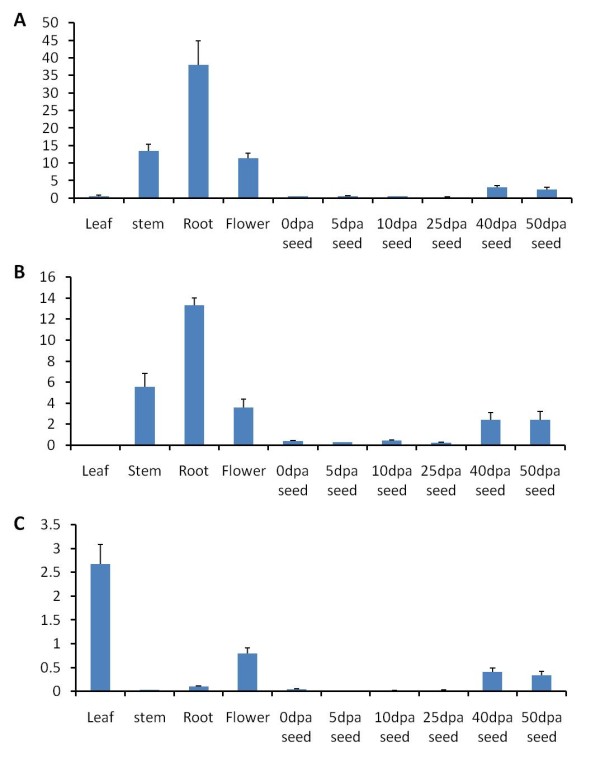
**GhCPS expression level in different tissues**. qRT-PCR analysis of putative cyclopropane synthase GhCPS1 (A), GhCPS2 (B) and GhCPS3 (C) among different tissues of cotton: leaf, stem, root, flower and seeds at different developing stages. The relative expression levels are reported relative to the expression of the ubiquitin 7 transcript. Data represents mean of triplicate measurements, error bar represents standard deviation.

To provide clues as to possible physiological roles of the three isozymes, their expression patterns in various tissues of cotton plants were examined. Quantitative reverse transcriptase (qRT)-PCR analysis of RNA from leaf, stem, root, flower and seeds at different development stages using gene-specific primers for the three isoforms revealed that GhCPS1 and 2 are highly expressed in root, stem, and flower. Both GhCPS1 and 2 showed low transcription levels in leaf and seeds at early development stages, i.e., from 0 to 25 day post anthesis (dpa) (Figure 2 A and B). In contrast, GhCPS3 showed highest transcription in leaf and flower but reduced levels in root and stem (Figure 2, C). All three GhCPS gene-transcript levels increased with seed development (Figure 2).

### Expression of GhCPS1 and 2 correlates with cyclic fatty acid accumulation levels

The FA profile of leaf, stem, root, flower and seeds at different development stages were determined to evaluate how they differed in their fatty acid compositions (Table 1). In root, stem and flower tissues, cyclic fatty acids made up about 19.2%, 9.9% and 4.0% of total fatty acids, respectively. Of these fatty acids, malvalic acid (7-(2-octyl-1-cyclopropenyl) heptanoic acid) was the most abundant, accounting for 11.9%, 6.9% and 3.0% of total fatty acids in root, stem and flower tissues, respectively. Cyclopropane and cyclopropene fatty acids were present at less than 2% in cotton leaf and seed tissues. With seed development, cyclic fatty acid increased to1.5% at 40 dpa seeds from less than 1.0% in 0, 5, 10 and 25 dpa seeds, and decreased to 1.2% in 50 dpa seeds.

**Table 1:** Tissue-specific FA composition of cotton tissues

	16:0%	16:1%	18:0%	18:1%	18:2%	18:3%	20:0%	MLV%	STC%	DHSA%
root	22.6 ± 0.16	0.4 ± 0.02	16.2 ± 0.43	11.7 ± 0.07	18.9 ± 0.41	10.1 ± 0.32	1.0 ± 0.03	11.9 ± 0.21	6.6 ± 0.13	0.7 ± 0.12
flower	20.7 ± 0.04	1.1 ± 0.01	7.1 ± 0.02	22.7 ± 0.01	20.3 ± 0.06	23.3 ± 0.13	0.8 ± 0.08	3.0 ± 0.02	0.9 ± 0.00	0.1 ± 0.09
seed 0dpa	23.9 ± 0.18	0.5 ± 0.05	11.0 ± 0.42	11.1 ± 0.43	36.5 ± 0.37	16.1 ± 0.34	0.5 ± 0.04		0.2 ± 0.02	0.4 ± 0.06
seed 5dpa	18.3 ± 0.24	0.3 ± 0.02	2.5 ± 0.04	19.6 ± 1.00	31.7 ± 0.37	26.8 ± 0.32	0.3 ± 0.06		0.3 ± 0.02	0.2 ± 0.02
seed 10dpa	20.1 ± 0.19	0.7 ± 0.04	4.8 ± 0.31	15.3 ± 0.72	26.8 ± 0.63	30.7 ± 0.50	0.5 ± 0.03		0.7 ± 0.01	0.4 ± 0.08
seed 25dpa	21.6 ± 0.43	0.6 ± 0.08	3.8 ± 0.21	13.9 ± 0.64	40.7 ± 0.52	18.4 ± 0.33	0.5 ± 0.17		0.3 ± 0.06	0.2 ± 0.06
seed 40dpa	20.3 ± 0.14	0.4 ± 0.02	2.2 ± 0.01	17.8 ± 0.05	53.3 ± 0.14	4.0 ± 0.07	0.4 ± 0.02	0.8 ± 0.01	0.3 ± 0.06	0.4 ± 0.08
seed 50dpa	21.0 ± 0.21	1.0 ± 0.03	2.5 ± 0.35	17.7 ± 0.12	54.4 ± 0.38	1.8 ± 0.25	0.5 ± 0.07	0.5 ± 0.07	0.3 ± 0.14	0.4 ± 0.09

Fatty acid composition from leaf, stem, root, flower and seeds at 0, 5, 10, 25, 40 and 50dpa were analyzed by gas chromatography/mass spectrometry. Results are expressed as a percentage of the total seed fatty acids, and 18:1 is the sum of 18:1Δ^9 ^and Δ^11 ^isomers. Values represent the mean and standard deviation of three replicates. MLV: malvalic adid; STC: sterculic acid; DHSA: dihydrosterculic acid.

In cotton tissues the abundance of GhCPS1 and 2 transcripts in different tissues is in general agreement with the cyclic fatty acid distribution, while GhCPS3 is expressed at very low levels in the root and stem tissues which are rich in the cyclic fatty acid. This suggests that GhCPS1 and 2 contribute to the CFA production in cotton. Cyclic fatty acids are synthesized very early in seed development, which was detected as early as 0 dpa in the ovule, and increased to peak at 40 dpa and then decreased a little at 50 dpa. This pattern is consistent with the expression pattern of GhCPS1, 2 and 3, but we cannot rule out GhCPS3 involvement in CFA production in the seed.

### Expression of GhCPS1 and 2 in yeast demonstrates their physiological activity

**Figure 3:**
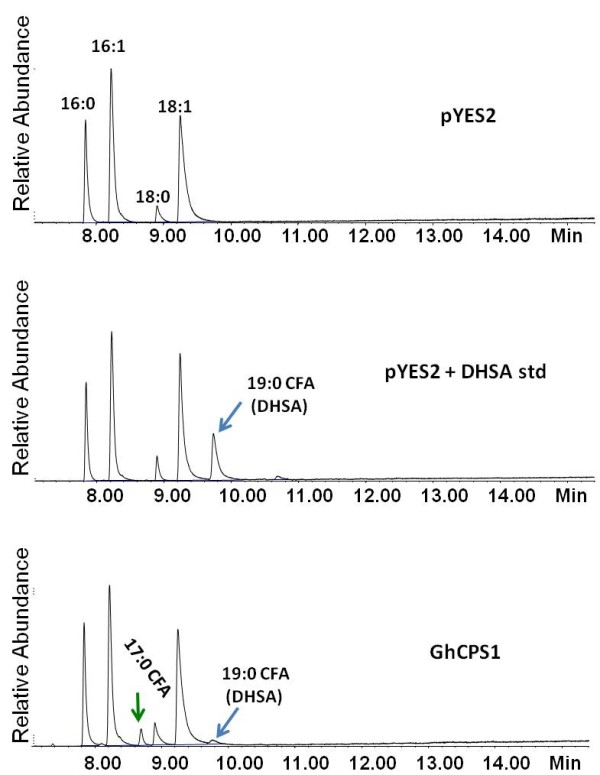
**GC analysis of FAMEs extracted from yeast expressing cotton CPS GhCPS 1**. After a 2-day induction with galactose (A) YES2; (B) YES2 spiked with DHSA and GhCPS1 which produced both 17:0 CFA and 19:0 CFA

**Figure 4:**
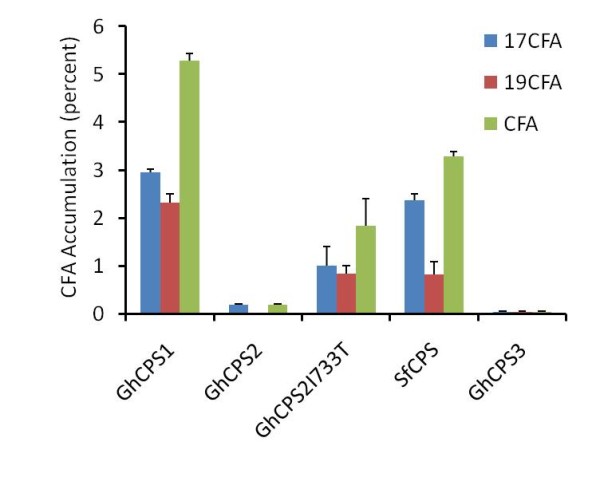
**Cyclopropane fatty acid production in CPS expressed yeast**. FAMEs were analyzed by GC/MS, both 17:0 CFA and 19:0 CFA were calculated as a percentage of the total fatty acids. The values represent the mean and standard deviation of three replicates.

To test whether the identified GhCPSs have enzymatic activity, the 3 genes were cloned into pYES2 vector and transformed into host strain YPH499. In addition to the authentic GhCPS2 sequence, a mutant, GhCPS2 I733T was identified and since the mutant point I733T is only one amino acid from the biocarbonate ion binding site, we decided to include it in our analysis. As shown in Figure 3, the fatty acid composition of yeast expressing GhCPS1 shows the production of two extra fatty acids relative to the control, the 17:0 CFA and 19:0 CFA. 17:0 CFA is identified by its mass ion from GC/MS. As shown in Figure 4, the GhCPS1 overexpression strain converted 16:1 FA to 2.96% of 17CFA and 18:1 FA into 2.32% of 19CFA, i.e., yielding a total of 5.28% CFA accumulation. The expression of GhCPS1 resulted in almost twice the CFA accumulation reported for the expression of the CPS from *Sterculia *[8]; SfCPS produced 2.36% of 17CFA and 0.82% of 19CFA, i.e., 3.18% total CFA. Expression of GhCPS2 resulted in only 0.36% CFA accumulation, while the expression of GhCPS3 didn't result in detectable levels of CFAs. Interestingly, the fortuitously obtained GhCPS2 I733T mutant resulted in the accumulation of 2.50% 17C and 19C cyclopropane, i.e., approximately 10-fold that of the WT GhCPS2. These results demonstrate that GhCPS 1 and 2 are indeed active CPSs that can act on both 16:1 and 18:1 fatty acid substrates to produce both 17C and 19C cyclopropane fatty acids.

### Deletion of the N-terminal oxidase domain decreases CPS activity

**Figure 5:**
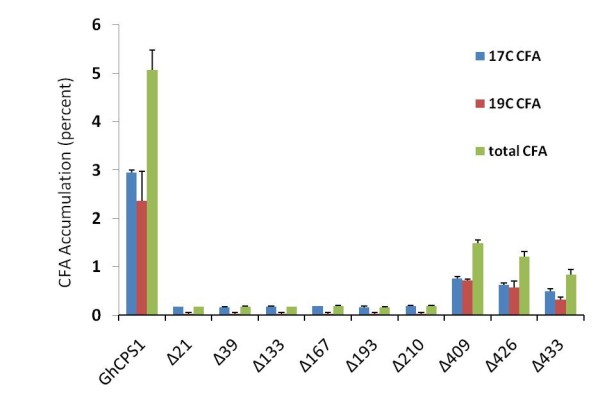
**Effect of N-terminal deletions on the CPS activity of GhCPS1**. Different portions of the N-terminal domain ranging from 21aa to 433 aa were deleted from GhCPS1, and their effects on CFA production analyzed. Both 17:0 and 19:0 CFAs were calculated as a percentage of the total fatty acids. The values represent the mean and standard deviation of three replicates.

Compared to bacterial CPS, GhCPS contain a ~400-amino acids-long N-terminal extension, homologous to oxidases that possesses an FAD-binding motif. In order to deduce the function of the oxidase domain of cotton CPS, different lengths of the N-terminal extension were deleted and the resulting constructs were expressed in yeast. After a 2-days of induction, a full length GhCPS1 yielded 2.94% 17CFA and 2.36% 19CFA, totaling 5.30% CFA. When the entire oxidase portion (409 aa) was deleted, the GhCPS1 still retained about 30% of the activity demonstrating that the oxidase activity is not necessary for CPS activity, but that it enhances CPS activity by an as yet unknown mechanism. Surprisingly, deletion of part of the oxidase domain, reduced CFA accumulation more than a total deletion (Figure 5), possibly by making the mRNA unstable or by incorrect folding of the protein, destabilizing it. Further deletion beyond the oxidase domain, i.e., into the N-terminal portion of the CPS domain, resulted in additional decreases in activity, with only 1.21% CFA accumulating upon deletion of 426 aa and 0.84% CFA upon deletion of 433 aa.

Co-expression of the *Agrobacterium *oxidase with the *E. Coli *CPS resulted in lower CFA accumulation in yeast compared to expression of the CPS alone. A similar decrease was found when the *Agrobacterium *oxidase was fused with *E. Coli *CPS protein. Fusion of a plant CPS N-terminal oxidase to *E.Coli *CPS also inhibited its ability to produce CFA (data not shown). These results demonstrate that unlike the cotton CPSs, *E.Coli *CPS activity is not enhanced by the oxidase domain. We cloned the *Agrobacterium*, gene AGR-C-3599p (N-terminal homolog to plant CPS) and AGR-C-3601p (C-terminal homolog to *plant CPS*) that was located 802 bp upstream of AGR-C-3599p. We also failed to detect any CFA from the ACPS over expression in yeast, and neither co-expression of these two proteins in yeast nor the fusion of these two polypeptides into a single polypeptide yielded any cyclopropane fatty acids (data not shown).

### Heterologous expression of GhCPS1 and 2 results in CFA accumulation in plants

**Figure 6:**
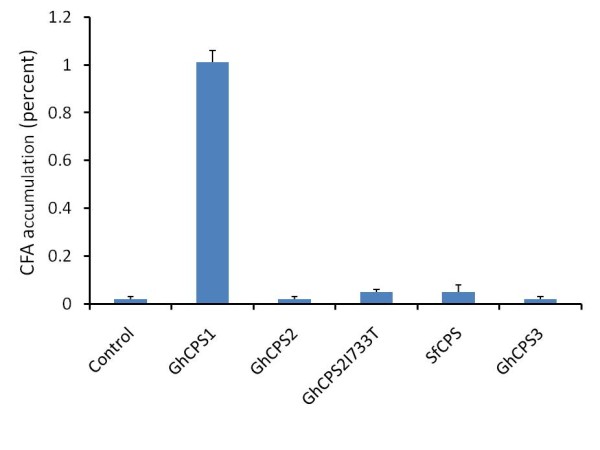
**Cyclopropane fatty acid production upon the expression of CPS in *fad2/fae1 *plants**. FAMEs were analyzed by GC/MS, cyclopropane fatty acid expressed as a percentage of the total fatty acids. The values represent the mean and standard deviation of three lines.

The CPS genes were transformed into Arabidopsis *fad2/fae1 *background with the GhCPS1 transgenic seeds yielding about 1.0% of 19C cyclopropane. No significant accumulation of cyclopropane products was detected in GhCPS2 and 3 over expression lines (Figure 6). Consistent with the GhCPS expression in yeast, a trace amount of cyclopropane was detected upon the expression of the SfCPS and GhCPS2 I733T mutant. Expression of CPSs in Arabidopsis seeds didn't lead to significant changes in other fatty acid composition and the oil content (data not shown).

When these genes were expressed in BY2 cell lines, ~1.0% of 19C cyclopropane was produced in GhCPS1 lines and SfCPS lines, and only a trace amount of CFA was detected in GhCPS2 transformed BY2 lines. No cyclopropane fatty acid was detected in lines transformed with GhCPS3, the chromatograms of which were indistinguishable from control pBI121-containing lines. In one of the 16 GhCPS2 I733T lines, 2.9% of CFA was detected.

These results demonstrate that GhCPS1 and 2 can cause the accumulation of CPA FA upon heterologous expression in plants.

## Discussion

Blast searching of the cotton genome database using the *Sterculia *CPS gene resulted in the identification of three cotton CPS homologous genes (GhCPS1, 2, 3). GhCPS1 and 2 show high similarities to the published SfCPS gene and their expression patterns correlate closely with CFA distributions in a variety of tissues. In addition, we confirmed their biochemical identity as cyclopropane synthases in yeast and plant.

Interestingly, GhCPS3's transcription level is relatively low in roots and stems where higher abundance of CFA is found. In addition, when heterologously expressed GhCPS3 did not result in detectable CFA accumulation in yeast, tobacco BY2 cell lines, or in Arabidopsis seeds. A number of plant sequences are related to GhCPS3; for instance, in Arabidopsis, 5 genes clustered in the same clade with GhCPS3 (Fig 1) and are more closely related to GhCPS3 than to SfCPS or to GhCPS 1 and 2. CPA and CPE have not been reported in Arabidopsis so far consistent with our analysis of Arabidopsis seeds and leaves in which we failed to identify any cyclopropane or cyclopropene fatty acids (data not shown). Since GhCPS3 and its Arabidopsis homologues are mainly expressed in leaf, it is possible that they catalyze the formation of other cyclopropanated products [9].

The role of the N-terminal oxidase portion of the plant-type CPS gene remains to be determined. From an evolutionary point of view, it is interesting to speculate on the origin of the cyclopropane synthases that contain the oxidase domain at the N-terminus. The oxidase gene and CPS gene are located adjacent to one another in the genomes of *Agrobacterium *and *Mycobacteria*; in plants the genes are fused to form a single product; taken together this suggests that the N-terminal domain in plants and its homologs in bacteria may play a role(s) related to cyclopropanation [9]. There is a conserved FAD-binding motif in the first 21 aa of the plant oxidase domain. It is hard to envisage how a redox system such as a FAD-containing protein could be involved in the catalytic reaction of methylene addition. After removal of the oxidase portion, the C-terminal CPS portion of GhCPS1 still retains 30% of its activity, showing that the oxidase activity is not necessary for function but that an intact oxidase domain somehow enhances activity perhaps by conferring stability to the CPS polypeptide. Cotton CPS antibodies would be helpful in distinguishing whether the reduction of activity upon partial, or complete deletion, of the oxidase domain results from destabilization of the enzyme or from loss of catalytic activity. It is possible that the oxidase portion plays a potential role in either the desaturation of dihydrosterculic acid to produce sterculic acid or the α-oxidation of the product to form malvalic acid.

Malvalic acid is a predominant CPE in cotton. No chain-shortened CPA was found when GhCPSs are expressed in yeast, tobacco BY2 cell lines, or *fad2/fae1 *Arabidopsis seeds. The data also show that the presence of CPA was insufficient to induce α-oxidation in these systems. Since neither CPE nor α-oxidation products were observed, we conclude that additional gene products are required for these functions.

A variety of bacteria initiate the cyclopropanation of fatty acids in the stationary phase or upon exposure to low pH [6,40,41], osmotic stress [42,43] and high temperature [44]. The conversion of unsaturated fatty acids into the corresponding CFAs reduces the levels of unsaturated fatty acids in membranes and therefore contributes to a reduction of the membrane fluidity which renders lipid bilayers more rigid [45]. Cyclization of fatty acid acyl chains is therefore generally regarded as a means to reduce membrane fluidity to adapt the cells for adverse conditions [6]. The content of cyclopropane fatty acids with 25 carbon atoms is correlated with early growth in spring for *Galanthus nivalis *L. and *Anthriscus silvestris *L. [46]. Lipids esterified with long chain cyclopropane fatty acids could contribute to the physiological adaptations of early spring plants and drought-tolerant plants by reducing membrane permeability to solvent [46].

CPE inhibited fatty acid desaturation in two fungi of interest to plant pathologists and CPE from *Sterculia foetida *affected *U. maydis*, the basidiomycete responsible for corn smut growth and morphology, suggesting that CPE serves as antifungal agent [47]. Study of gene expression changes in *Fusarium oxysporum *f. sp. *vasinfectum*-infected cotton seedlings identified GhCPS2 (i.e., CD486555) as having increased expression in cotton roots at 3 days post-infection, together with a bacterially induced lipoxygenase. This makes GhCPS2 one of the few potential defense-related genes induced in infected roots and putative stress-related genes encoding proteins such as glutathione S-transferase (GST) 18 and nitropropane dioxygenase [48].

## Conclusions

We have shown that both GhCPS1 and 2 contribute to CFA accumulation in cotton seeds; but GhCPS1 accounts for the majority of CFA accumulation in roots and stems. The information presented herein has potential uses for two distinct biotechnological applications. It is highly desirable to target both GhCPS1 and 2 for suppression to reduce the CPE content of cottonseed meal for use as animal feed. Conversely, to facilitate CPA accumulation for use as oleochemical feedstocks, our data suggests GhCPS1 to be the best choice for heterologous expression in a production plant.

## Authors' contributions

JS conceived of and provided the initial design of the study. X-HY and RR performed the research. All authors contributed to the manuscript preparation, and read and approved the final manuscript.

## Supplementary Material

Additional file 1 **Sequence alignment of CPS**. Amino acid sequence alignment of CPS from different organisms Click here for file

Additional file 2 **qPCR experiments/MIQE**. Minimum Information for Publication of Quantitative Real-Time PCR Experiments Click here for file
