# Supplementary material for: Characterization and analysis of the cotton cyclopropane fatty acid synthase family and their contribution to cyclopropane fatty acid synthesis
Source: BMC Plant Biol. 2011 May 25;11:97. doi: 10.1186/1471-2229-11-97 (PMC3132707; doi:10.1186/1471-2229-11-97)
Supplement: Additional file 1 — Sequence alignment of CPS. Amino acid sequence alignment of CPS from different organisms [file 1471-2229-11-97-S1.DOCX]

**Supplementary Figure 1**

**Amino acid sequence alignment of CPS from different organisms.**

AT3G23510 MKVAVIGSGISGLGSAYVLANQGVKEVVLYEKEESLGGHAKTVRFDGVDLDLGFMVFNRV 60

AT3G23530 MKVAVIGSGISGLGSAYVLANQGVKEVVLYEKEESLGGHAKTVRFDGVDLDLGFMVFNRV 60

AT3G23470 ------------------------------------------------------------

AT3G23480 ------------------------------------------------------------

AT3G23460 ------------------------------------------------------------

GhCPS3 MKIAVIGGGISGVVSAYTLAKAGAN-VVLYEKEEYLGGHSKTVHFDGVDLDLGFMVFNRV 59

GhCPS1 MEVAVIGGGIKGLLSAYVLVKAGVD-VVVYEKEEQLGGHAKTVNFDAVDLDLGFLFLNPA 59

GhCPS2 MEVAVIGGGIKGLVSAYVLVKAGVD-VVVYEKEEQLGGHAKTVNFDAVDLDLGFLFLNPA 59

SfCPS MGVAVIGGGIQGLVSAYVLAKAGVN-VVVYEKEEQVGGHAKTVSFDAVDLDLGLLFLNPA 59

AtCPS ------------------------------------------------------------

cmaA1 ------------------------------------------------------------

mmaA2 ------------------------------------------------------------

pcaA ------------------------------------------------------------

cmaA2 ------------------------------------------------------------

EcCPS ------------------------------------------------------------

AT3G23510 TYPNMIEFFENLGVEMEVSDMSFAVSLDNGK-GCEWGSRNGVSGLFAQKKNVLNPYFWQM 119

AT3G23530 TYPNMMEFFENLGVEMEVSDMSFAVSLDNGK-GCEWGSRNGVSGLFAQKKNVLNPYFWQM 119

AT3G23470 ------------------------------------------------------------

AT3G23480 ------------------------------------------------------------

AT3G23460 ------------------------------------------------------------

GhCPS3 TYPNMMELFESLGIDMEPFDMSLSVSLNEGK-GCEWGSRNGLSALFAQKSNLFNPYFWQM 118

GhCPS1 RYATLLHMFDSLGVDVETSDVSFSISHDKGNNGYEWCSQYGFSNYFAQKKKLLNPFNWQS 119

GhCPS2 RYATLLDIIDSLGVDVETSDVSFSISHDKGNNGYEWCSQYGFSNYFAQKKKLLNPFNWQN 119

SfCPS RYPTMLELFDSLEVDVEATDVSFSVSHDKGN-GYEWCSQYGFSNFLAHKKKMLNPYNWQD 118

AtCPS ------------------------------------------------------------

cmaA1 ------------------------------------------------------------

mmaA2 ------------------------------------------------------------

pcaA ------------------------------------------------------------

cmaA2 ------------------------------------------------------------

EcCPS ------------------------------------------------------------

AT3G23510 IREIVRFKEDVLNYIEKLEGNPDIDRKETLGEFLNTRGYSELFQQAYLVPICGSIWSCPS 179

AT3G23530 IREIVRFKEDVLKYIEELEGNPDIDRKETLGEFLNSRGYSELFQQAYLVPICGSIWSCPS 179

AT3G23470 ------------------------------------------------------------

AT3G23480 ------------------------------------------------------------

AT3G23460 ------------------------------------------------------------

GhCPS3 LREILKFKNDVISYLELLENNPDIDRNETLGQFIKSKGYSDLFQKAYLVPVCGSIWSCPT 178

GhCPS1 LREIIKFGNDVESYLGSLENNPDIDRTETLGQFINSKGYSENFQNTYLAPICGSMWSSSK 179

GhCPS2 LREIIRFSNDVESYLGSLENNPDIDRTETLGQFIKSKGYSENFQNTYLAPICGSMWSSSK 179

SfCPS LRETIKFGNDVNSYLESLEKNPDIDRNETLGHFVGSKGYSENFLNTYLAPICGSMWSCSK 178

AtCPS ------------------------------------------------------------

cmaA1 ------------------------------------------------------------

mmaA2 ------------------------------------------------------------

pcaA ------------------------------------------------------------

cmaA2 ------------------------------------------------------------

EcCPS ------------------------------------------------------------

AT3G23510 DGVLSFSAYSVLSFCCNHHLLQIFGRPQWLTVAGRSQTYVAKVRAELERLGCKIRTSCDV 239

AT3G23530 DGVLSFSAYSVLSFCCNHHLLQIFGRPQWLTVAGRSQTYVAKVRTELERLGCRIRTSCDV 239

AT3G23470 ------------------------------------------------------------

AT3G23480 ------------------------------------------------------------

AT3G23460 ------------------------------------------------------------

GhCPS3 ERVMDFSAFSILSFCRNHHLLQIFGRPQWMTVRWRSHRYVNKVREELESTGCQIRTGCEV 238

GhCPS1 EDVTSFSAFSILSFCRTHHLYQLFGQSQWLTIKGHSH-FVKRVREVLETKGCQFKLGCEV 238

GhCPS2 EDVMSFSAFSILSFCRTHHLYQQFGQPQWLTIKGHSH-FVKRVREVLETKGCQFKLGCEV 238

SfCPS EEVMSFSAYSILSFCRTYHLYQLFGNPQWLTIKRHSY-LVKKVRDILESRGCQFKLGCEV 237

AtCPS ------------------------------------------------------------

cmaA1 ------------------------------------------------------------

mmaA2 ------------------------------------------------------------

pcaA ------------------------------------------------------------

cmaA2 ------------------------------------------------------------

EcCPS ------------------------------------------------------------

AT3G23510 KSVSTSENGCVTVTSGDGSEEVFDRCILAMHAPDALRLLGEEVTFDESRVLGAFQYVYSD 299

AT3G23530 KSVSTSENGCVTVTSGDGSKEVFDRCILAMHAPDALRLLGEEVTFDESRVLGAFQYVYSD 299

AT3G23470 ------------------------------------------------------------

AT3G23480 ------------------------------------------------------------

AT3G23460 ------------------------------------------------------------

GhCPS3 HSVLSDAEG-CTVLCGDDSHELYQGCIMAVHAPYALRLLGNQATYDESTVLGAFQYVYSD 297

GhCPS1 QSVLPVDNG-TAMVCGDGFQETYNGCIMAVDAPTALKLLGNQATFEETRVLGAFQYATSD 297

GhCPS2 QSVLPADNG-TTMVCGDGFQETYNGCIMAVDAPTALKLLGNQATFEETRVLGAFQYATSD 297

SfCPS LSVLPADDG-SSIVFGDGFQETYNGCIMAVNAPTALKILGNQATFEEMRVLGAFQYASSD 296

AtCPS ------------------------------------------------------------

cmaA1 ------------------------------------------------------------

mmaA2 ------------------------------------------------------------

pcaA ------------------------------------------------------------

cmaA2 ------------------------------------------------------------

EcCPS ------------------------------------------------------------

AT3G23510 IYLHHDIDLMPRNKAAWSAWNFLGSTEKKVCVTYWLNILQNLGENSEPFFVTLNPDETPK 359

AT3G23530 IYLHHDIDLMPRNQAAWSAWNFLGSTEKKVCVTYWLNILQNLGENSEPFFVTLNPDETPK 359

AT3G23470 ------------------------------------------------------------

AT3G23480 ------------------------------------------------------------

AT3G23460 ------------------------------------------------------------

GhCPS3 IYLHRDKNLMPKNPAAWSAWNFLGSTDKNVSLTYWLNVLQNLGETSLPFLVTLNPDYTPK 357

GhCPS1 IFLHQDSTLMPQNKSAWSALNFLNSSKNNAFLTYWLNALQNIGKTSEPFFVTVNPDHTPK 357

GhCPS2 IFLHRDSTLMPQNKSAWSALNFLNSSKNNAFLTYWLNALQNIGKTSEPFFVTVNPDHTPK 357

SfCPS IYLHRDSNLMPTNRSGWSALNFLRSRENKASLTYWLNVLQNVGKTSQPFFVTLNPDRIPD 356

AtCPS ------------------------------------------------------------

cmaA1 ------------------------------------------------------------

mmaA2 ------------------------------------------------------------

pcaA ------------------------------------------------------------

cmaA2 ------------------------------------------------------------

EcCPS ------------------------------------------------------------

AT3G23510 KALLKWTTGHPVPSVAASIASQELHQIQGKRNIWFCGAYQGYGFHEDGLKAGMAAARGLL 419

AT3G23530 KTLLKWTTGHPVPSVAAWTASQELHKIQGKRNIWFCGAYQGYGFHEDGLKAGMAAARGLL 419

AT3G23470 ----------------------------------------------------MVIAQSLL 8

AT3G23480 ----------------------------------------------------MVIARGLL 8

AT3G23460 ------------------------------------------------------------

GhCPS3 HTLLKWRTGHPVPSVAATKASLELDRIQGKRGIWFCGAYLGYGFHEDGLKAGMIAANGLL 417

GhCPS1 NTLLKWSTGHAIXSVAASKASLELGQIQGKRGIWFCG----YDFNQDELKAGMDAAHGIL 413

GhCPS2 NTLLKWSTGHAIPSVAASKASLELGQIQGKRGIWFCG----YDFNQDELKAGMDAAHGIL 413

SfCPS KILLKWSTGRPIPSVAASKASLELDQIQGKRGIWFCG----YDFHEDELKAGMDAAHRIL 412

AtCPS ----------------------------------------------------MTPFENLS 8

cmaA1 ------------------------------------------------------------

mmaA2 ------------------------------------------------------------

pcaA ------------------------------------------------------------

cmaA2 ------------------------------------------------------------

EcCPS ------------------------------------------------------------

AT3G23510 GKETALLNNPR--------HMVPSLTETGARLFVTRFLGQFISTGSVTILEEGGTMFTFG 471

AT3G23530 GKETALLNNPR--------HMVPSLTETGARLFVTRFLGQFISTGSVTILEEGGTMFTFG 471

AT3G23470 WKEITLLKKIQ--------HMVLSLTESGARLFVTRFLEQFISIGCVTILEEGGTMFIFG 60

AT3G23480 GIQTTLVKNMQ--------HMVFSLTEAGARLFVTRFFRQFISIGCVTIL-EGDTIFIFG 59

AT3G23460 --MVTPMSNRK--------HMVMSLIEKAARFFFTRFLTHFISTGCVTIF-EGGNMVTFE 49

GhCPS3 GKSCNILSNPK--------HMVPSLMETGARLFVTRFLSHFISTGCVILLEEGGTMFTFE 469

GhCPS1 GKHSSVPPSPKNMSPSLPKNMSPSFMETTARLFVTKFFQQYISMGCVIFLEEGGRIFTFK 473

GhCPS2 GKHSSVLHSPK--------SMSPSFMETTARLFVTKFFQQYISMGCVIFLEEGGRIFTFK 465

SfCPS GKHFSVLHSPR--------QMSPSFMETTARLLVTKFFHQYIQVGCVIIIEEGGRVYTFK 464

AtCPS ELPAQNTRFNG---------------RAGMGTWLVNRLLNNIERGRLRVTLPGGGTIEKS 53

cmaA1 ------------------------------------------------------------

mmaA2 ------------------------------------------------------------

pcaA ------------------------------------------------------------

cmaA2 ------------------------------------------------------------

EcCPS ---------------------------------MSSSCIEEVSVPDDNWYRIANELLSRA 27

AT3G23510 GKDSTCPLKSILKIHSPQFYWKVMTQADLGLADAYINGDFSFVDKESGLLNLIMILIANR 531

AT3G23530 GKDSTCPLKSILKIHSPQFYWKVMTQADLGLADAYINGDFSFVDKESGLLNLIMILIANR 531

AT3G23470 EKDSTCPLKSILKIHSPQFYWKVMTQADLGLADAYISGDFSFVDKDSGLLNLIMILIANR 120

AT3G23480 ENESTCPLKSILKIHSPQFYWKVMTLADLGLADAYINGDFSFVDKDSGLLNLIMILIANR 119

AT3G23460 GKDSRCHLKSELEIHSPQFYWKVMTQVDLGLADAYINGDFSFVNKETGLLNLIMILIASK 109

GhCPS3 GTSNKCSLKTVIKVHSPHFYWKVMTEADLGLADSYINGDFSFVDKKDGLLNLVMILIANR 529

GhCPS1 GNMEKCPLKTVLKVHNPQFYWRIMKEADIGLADAYIHGDFSFLDENEGLLNLFRILVANK 533

GhCPS2 GNMEKCPLKTVLKVHNPQFYWRIMKEADIGLADAYIHGDFSFLDETEGLLNLFRILVANK 525

SfCPS GSMENCSLKTALKVHNPQFYWRIMKEADIGLADAYIQGDFSFVDKDDGLLNLFRILIANK 524

AtCPS GHQEGS--EAILMLHNWRAIRRVLVNGDIGFAEGFIENDWSTPD----LTALIRFAAQNQ 107

cmaA1 ------------------------------------------------MP---------- 2

mmaA2 ------------------------------------------------MV---------- 2

pcaA ------------------------------------------------MS---------- 2

cmaA2 ------------------------------------------------MTSQGDTTSG-- 10

EcCPS GIAINGSAPADIRVKNPDFFKRVLQEGSLGLGESYMDGWWECDRLDMFFSKVLRAGLE-- 85

:

AT3G23510 DTK---SNLTKKRGWWTPMFLTAGLASAKYFLKHVSRQNTLTQARRNISRHYDLSNELFG 588

AT3G23530 DTK---SNLSKKRGWWTPMFLTAGLASAKYFLKHVSRQNTLTQARRNISRHYDLSNELFG 588

AT3G23470 DQRSPKSNLVKKRGWWTPVFLTAGLASVKYYLKHVLKQNTLTQARKNISSHYDLSNEFFG 180

AT3G23480 DLNSRKSNLAKKRGWWTPVFLTASLASATYYLKHVCRQNTLTQARRNVSSHYDLSNEFFG 179

AT3G23460 ELN---SNLAEKRGRWTPIFLTTGLSSAKHFLKHLYRQNNLTQARRNISRHYDLSNELFT 166

GhCPS3 DLISSNSKLSKKRGWWTPLLFTAGLTSAKYFFKHVLRQNTLTQARRNISRHYDLSNDLFA 589

GhCPS1 ENS-AASGSTKRRTWWSPALLTASISSAKYFVKHLLRQNTITQARRNISRHYDLSNELFS 592

GhCPS2 ENS-AASGSNKRRTWWSPALLTASISSAKYFVKHLLRQNTITQARRNISRHYDLSNELFT 584

SfCPS ELN-SASGQNKRRTWLSPALFTAGISSAKYFLKHYMRQNTVTQARRNISRHYDLSNELFT 583

AtCPS DAF-----ARSTRGSLPMRLINR--------MAHALNANTRRGSRRNIEAHYDLGNEFYR 154

cmaA1 -------------DELKPHFANVQ-------------------------AHYDLSDDFFR 24

mmaA2 -------------NDLTPHFEDVQ-------------------------AHYDLSDDFFR 24

pcaA -------------VQLTPHFGNVQ-------------------------AHYDLSDDFFR 24

cmaA2 -------------TQLKPPVEAVR-------------------------SHYDKSNEFFK 32

EcCPS -------------NQLPHHFKDTLRIAGARLFNLQSKKRAWIVGK----EHYDLGNDLFS 128

. *** .::::

AT3G23510 LFLDDTMTYSSAVFKSDDEDLRTAQMRKISLLIDKARIEKDHEVLEIGCGWGTLAIEVVR 648

AT3G23530 FFLDDTMTYSSAVFKSDDEDLRTAQMRKISLLIDKARIEKDHEVLEIGCGWGTLAIEVVR 648

AT3G23470 LFMDDTMMYSSAIFKSENEDPRTAQMRKISLLIEKARIEKNHEVLEMGCGWGTFAIEVVK 240

AT3G23480 LFMDDTMMYSSAVFKSENENLRTAQMRKIHLLIEKDKEEP-YEVLEIGCGWGTLAIEVVK 238

AT3G23460 IFLDDTMSYSSGVFKSDDEELKIAQMRKIYLLIEK------------------------- 201

GhCPS3 LFLDETMTYSCAVFKTEDEDLKDAQHRKISLLIEKARIDSKHEILEIGCGWXSLAIEVVK 649

GhCPS1 LYLGKMMQYSSGVFRTGEEHLDVAQRRKISSLIEKTRIEKWHEVLDIGCGWGSLAIETVK 652

GhCPS2 LYLGKMMQYSSGVFRTGEEHLDVAQRRKISSLIEKARIEKRHEVLDIGCGWGSLAIETVK 644

SfCPS LYLGEMMQYSSGIFKTGEEHLDVAQRRKISSLIDKSRIEKWHEVLDIGCGWGSLAMEVVK 643

AtCPS QWLDPSMLYSSAIFDDTTPTLEAAQRKKLERIAEKLQLTGNNSVLEIGCGWGALAIHLAT 214

cmaA1 LFLDPTQTYSCAYFERDDMTLQEAQIAKIDLALGKLGLQPGMTLLDVGCGWGATMMRAVE 84

mmaA2 LFLDPTQTYSCAHFEREDMTLEEAQIAKIDLALGKLGLQPGMTLLDIGCGWGATMRRAIA 84

pcaA LFLDPTQTYSCAYFERDDMTLQEAQIAKIDLALGKLNLEPGMTLLDIGCGWGATMRRAIE 84

cmaA2 LWLDPSMTYSCAYFERPDMTLEEAQYAKRKLALDKLNLEPGMTLLDIGCGWGSTMRHAVA 92

EcCPS RMLDPFMQYSCAYWKDADN-LESAQQAKLKMICEKLQLKPGMRVLDIGCGWGGLAHYMAS 187

:. **.. : ** * *

AT3G23510 RTGCKYTGITLSIEQLKYAEEKVKEAGLQDRITFELRDYRQLSDAHKYDRIISCEMLEAV 708

AT3G23530 RTGCKYTGITLSIEQLKYAEEKVKEAGLQDWITFELRDYRQLSDAQKYDRIISCEMLEAV 708

AT3G23470 RTGCKYTGITLSIEQLKYAKAKVKEAGLQGRITFMLCDYRQLSDARKYDRIIACEMIEAV 300

AT3G23480 RTGCKYTGFTLSIEQLKYVEEKVKEAGLQERITFKLCDYRQLCDTQKYDRIISCEMIEHV 298

AT3G23460 ------------------------------------------------------------

GhCPS3 RTGCKYTGITLSEEQLKLAEKRVKEAGLQENIRFQLCDYRQLPSTYKYDRIISCEMIEAV 709

GhCPS1 RTGCKYTGITLSEQQLKYAQEKVKEAGLEDNIKILLCDYRQLPKEHQFDRIISVEMVEHV 712

GhCPS2 RTGCKYTGITLSEQQLKYAQEKVKEAGLQDNIKILLCDYRQLPKEHQFDRIISVEMVEHV 704

SfCPS RTGCKYTGITLSEQQLKYAEEKVKEAGLQGNIKFLLCDYRQLPKTFKYDRIISVEMVEHV 703

AtCPS QQNADVTGITLSPSQLRWAENAAEKESKAGRIDLRLQDYRDVQG--QFDNIVSVEMFEAV 272

cmaA1 KYDVNVVGLTLSKNQANHVQQLVANSENLRSKRVLLAGWEQFDEP--VDRIVSIGAFEHF 142

mmaA2 QYDVNVVGLTLSKNQAAHVQKSFDEMDTPRDRRVLLAGWEQFNEP--VDRIVSIGAFEHF 142

pcaA KYDVNVVGLTLSENQAGHVQKMFDQMDTPRSRRVLLEGWEKFDEP--VDRIVSIGAFEHF 142

cmaA2 EYDVNVIGLTLSENQYAHDKAMFDEVDSPRRKEVRIQGWEEFDEP--VDRIVSLGAFEHF 150

EcCPS NYDVSVVGVTISAEQQKMAQERCEGLDVT----ILLQDYRDLNDQ--FDRIVSVGMFEHV 241

AT3G23510 GH-------EFMEMFFSRCEAALAEDGLMVLQFISTPEER---------YNEYRLSSDFI 752

AT3G23530 GH-------EFMEMFFSRCEAALAENGLIVLQFISIPEER---------YNEYRLSSDFI 752

AT3G23470 GH-------EFMDKFFSCCEDALAENGIFVLQFTAIPEAL---------YDESRLTSGFI 344

AT3G23480 GH-------KFMETFFSHCEAALAEDGIFVLQFTAIPEEL---------YDESRLTSGFI 342

AT3G23460 ------------------------------------------------------------

GhCPS3 GH-------EYMEDFFGCCESVLADDGLLVLQFISIPEER---------YNEYRRSSDFI 753

GhCPS1 GE-------EYIEEFYRCCDQLLKEDGLFVLQFISIPEEL---------SKEIQQTAGFL 756

GhCPS2 GE-------EYIEEFYRCCDQLLKEDGLFVLQFISIPEEL---------SKEIQQTAGFL 748

SfCPS GE-------EYIEEFFRCCDSLLAENGLFVLQFISIPEIL---------SKEIQQTAGFL 747

AtCPS GE-------SYWPSYFEMLKRCLKPGGRAVLQIISIDESR---------FDTYRRKADFI 316

cmaA1 GH-------ERYDAFFSLAHRLLPADGVMLLHTITGLHPKEIHERGLPMSFTFARFLKFI 195

mmaA2 GH-------DRHADFFARAHKILPPDGVLLLHTITGLTRQQMVDHGLPLTLWLARFLKFI 195

pcaA GH-------QRYHHFFEVTHRTLPADGKMLLHTIVRPTFKEGREKGLTLTHELVHFTKFI 195

cmaA2 ADGAGDAGFERYDTFFKKFYNLTPDDGRMLLHTITIPDKEEAQELGLTSPMSLLRFIKFI 210

EcCPS GP-------KNYDTYFAVVDRNLKPEGIFLLHTIGSKKTDLNVDP-------------WI 281

AT3G23510 KEYIFPGACVPSLAKVTSAMSSSSRLCIEHVENIGIHYYQTLRLWRKNFLERQKQIMALG 812

AT3G23530 KEYIFPGGCLPSLARVTTAMSSSSRLCIEHVENIGIHYYQTLRLWRKNFLARQKQIMALG 812

AT3G23470 TEYIFPGGCLPSLARVTSAMASSSRLCIENVENIGIHYYHTLRCWRKNFLERQKQIIDLG 404

AT3G23480 TEYIFPGGCLPSLARVTSAMASSSRLCIENVENIGIHYYPTLRYWRKNLLERQKQIIDLG 402

AT3G23460 -----------------TAYLSCS---IENVENIGIHYYQTLRLWRKNFFERQKQITDLG 241

GhCPS3 KEYIFPGGCLPSLARITTAMNAASKLCVEHVENIGLHYYQTLRYWRKNFLEKQSKIHALG 813

GhCPS1 KEYIFPGGTLLSLDRNLSAMAAATRFSVEHVENIGMSYYHTLRWWRKLFLKNTSKVLALG 816

GhCPS2 KEYIFPGGTLLSLDRNLSAMAAATRFSVEHVENIGMSYYHTLRWWRKLFLENTSKVLALG 808

SfCPS KEYIFPGGTLLSLDRTLSAMAAASRFSVEHVENIGISYYHTLRWWRKNFLANESKVLALG 807

AtCPS QKYVFPGGFLPSDSALEKSVGQAG-LKLTETELFGQSYALTLAEWRQRFHARWQTISLLG 375

cmaA1 VTEIFPGGRLPSIP-MVQECASANGFTVTRVQSLQPHYAKTLDLWSAALQANKGQAIAL- 253

mmaA2 ATEIFPGGQPPTIE-MVEEQSAKTGFTLTRRQSLQPHYARTLDLWAEALQEHKSEAIAI- 253

pcaA LAEIFPGGWLPSIP-TVHEYAEKVGFRVTAVQSLQLHYARTLDMWATALEANKDQAIAI- 253

cmaA2 LTEIFPGGRLPRIS-QVDYYSSNAGWKVERYHRIGANYVPTLNAWADALQAHKDEAIAL- 268

EcCPS NKYIFPNGCLPSVR-QIAQ-SSEPHFVMEDWHNFGADYDTTLMAWYERFLAAWPEIADN- 338

: . : * ** * :

AT3G23510 FDDKFVRTWEYYFDYCAAGFKTRTLGDYQLVFSRPGNVAAFADSYRGFPSAYCVS----- 867

AT3G23530 FDDKFVRTWEYYFDYCAAGFKTLTLGNYQLVFSRPGNVAAFADSYRGFPSAYCVT----- 867

AT3G23470 FDDKFIRTWEYYFDYCAAGFKTLTLRSYQIVFSRPGNVAAFGDDDPFRSSTR-------- 456

AT3G23480 FDEKFLRTWEYYFDYCAAGFKTLTLRNYQIVFSRPGNVAAFGNDDPFHSSALTQEMGLIL 462

AT3G23460 FDDRFVRTCEYYFDYCAAGFKTRTVGDYQIVFSRPGNVIALG-DDSFYSSPLTQ------ 294

GhCPS3 FNDKFIRTWEYYFDYCAAGFKSNTLGNYQVVFSRPGNVVALGNPYKDFPSAS-------- 865

GhCPS1 FDEKFMRTWEYYFDYCAAGFKTGTLIDYQVVFSRAGNFGTLGDPYKGFPSAYSFMDD--- 873

GhCPS2 FDEKFMRTWEYYFDYCAAGFKTGTLIDYQVVFSRAGNFGTLGDPYKGFPSAYSFMDD--- 865

SfCPS FDEKFMRTWEYYFDYCAAGFKTGTLIDYQVVFSRAGNFAALGDPYIGFPSAYSYSDN--- 864

AtCPS FDERFRRLWDYYLCYCEAGFAEGTINVGLYTIEHG------------------------- 410

cmaA1 QSEEVYERYMKYLTGCAEMFRIGYIDVNQFTCQK-------------------------- 287

mmaA2 QSEEVYERYMKYLTGCAKLFRVGYIDVNQFTLAK-------------------------- 287

pcaA QSQTVYDRYMKYLTGCAKLFRQGYTDVDQFTLEK-------------------------- 287

cmaA2 KGQETYDIYMHYLRGCSDLFRDKYTDVCQFTLVK-------------------------- 302

EcCPS YSERFKRMFTYYLNACAGAFRARDIQLWQVVFSRGVENGLRVAR---------------- 382

.: *: * * . :

AT3G23510 ---------------------------------------------------

AT3G23530 ---------------------------------------------------

AT3G23470 -----KNNII----------------------------------------- 461

AT3G23480 QTGNVSSNVVFSVCFPLSFIICKRLPRHLKQFCFHDYAFCSNNLSLSRPYI 513

AT3G23460 -----KKQLQMNHFDS----------------------------------- 305

GhCPS3 ---------------------------------------------------

GhCPS1 ---------------------------------------------------

GhCPS2 ---------------------------------------------------

SfCPS ---------------------------------------------------

AtCPS ---------------------------------------------------

cmaA1 ---------------------------------------------------

mmaA2 ---------------------------------------------------

pcaA ---------------------------------------------------

cmaA2 ---------------------------------------------------

EcCPS ---------------------------------------------------
