# Supplementary material for: Characterization and analysis of the cotton cyclopropane fatty acid synthase family and their contribution to cyclopropane fatty acid synthesis
Source: BMC Plant Biol. 2011 May 25;11:97. doi: 10.1186/1471-2229-11-97 (PMC3132707; doi:10.1186/1471-2229-11-97)
Supplement: Additional file 2 — qPCR experiments/MIQE. Minimum Information for Publication of Quantitative Real-Time PCR Experiments [file 1471-2229-11-97-S2.DOC]

| **EXPERIMENTAL DESIGN** |
| --- |
| Definition of experimental and control groups |
| Number within each group |
| Assay carried out by core lab or investigator's lab? |
| Acknowledgement of authors' contributions |
| **SAMPLE** |
| Description |
| Volume/mass of sample processed |
| Microdissection or macrodissection |
| Processing procedure |
| If frozen - how and how quickly? |
| If fixed - with what, how quickly? |
| Sample storage conditions and duration (especially for FFPE samples) |

Experimental design is provided in the material and method section. RNA was isolated from cotton leaf, flower, stem, root and seeds of 0, 5, 10, 25, 40, 50 dpa. Assays were carried out in the investigator’s lab.

In general, about 2-5 g of leaf, root, stem and flower tissues and 0.5-2 g of seeds from different development stages were collected and flashed frozen with liquid nitrogen and stored in -80°C until use.

| **NUCLEIC ACID EXTRACTION** |
| --- |
| Procedure and/or instrumentation |
| Name of kit and details of any modifications |
| Source of additional reagents used |
| Details of DNase or RNAse treatment |
| Contamination assessment (DNA or RNA) |
| Nucleic acid quantification |
| Instrument and method |
| Purity (A260/A280) |
| Yield |
| RNA integrity method/instrument |
| RIN/RQI or Cq of 3' and 5' transcripts |
| Electrophoresis traces |
| Inhibition testing (Cq dilutions, spike or other) |

RNA from leaf, flower, stem and seeds at different development stages were extracted according to Wu et al. (2002) and RNA from cotton root was isolated using Qiagen’s plant Mini RNA kit (Cat#74903). For efficient elimination of genomic DNA contamination from starting RNA samples, gDNA wipeout buffer from Qiagen’s QuanTect Reverse Transcription Kit (Cat#203511) was used as manufacture’s protocol. Contamination was assessed by a no RT control.RNA quality and concentration were determined by gel electrophoresis and Nanodrop spectroscopy, the A260/280 ratio is generally between 1.9 and 2.1, exact values of each RNA can be provided upon request. The yield ranged from 0.5-2 mg of RNA per 200 mg of tissues.

RNA intergrity (RIN/RQI or Cq of 3/ and 5’ transcripts) and inhibition testing was not performed.

(Wu YR, Llewellyn DJ, Dennis ES: **A quick and easy method for isolating good-quality RNA from cotton (Gossypium hirsutum L.) tissues.** *Plant Mol Biol Rep* 2002, **20:**213-218.)

| **REVERSE TRANSCRIPTION** |
| --- |
| Complete reaction conditions |
| Amount of RNA and reaction volume |
| Priming oligonucleotide (if using GSP) and concentration |
| Reverse transcriptase and concentration |
| Temperature and time |
| Manufacturer of reagents and catalogue numbers |
| Cqs with and without RT |
| Storage conditions of cDNA |

Reverse transcription (RT) was carried out using Qiagen’s QuanTect Reverse Transcription Kit. (Cat#205311) in a 20 µl reaction volume. Add 0.5 µg of RNA, 2µl of gDNA Wipeout Buffer (7x), and RNase-free water to a total volume of 14 µl, incubate for 2 min at 42°C. Then place immediately on ice. Add template RNA to tube containing 1 µl of Quantiscript Reverse Transcriptase, 4 µl of Quantiscript RT Buffer and 1 µl of oligo(dT)_18_ mix, incubate 30 min at 42°C and then incubate at 95°C to inactivate Quantiscript Reverse Transcriptase. For most cDNAs, the *ubiq7* amplicon was used for the estimation of contamination, without RT no amplification was detected. cDNA was stored in low adhesion tubes at -20°C.

| **qPCR TARGET INFORMATION** |
| --- |
| If multiplex, efficiency and LOD of each assay. |
| Sequence accession number |
| Location of amplicon |
| Amplicon length |
| *In silico* specificity screen (BLAST, etc) |
| Pseudogenes, retropseudogenes or other homologs? |
| Sequence alignment |
| Secondary structure analysis of amplicon |
| Location of each primer by exon or intron (if applicable) |
| What splice variants are targeted? |

Multiplex qPCR was not performed. Sequence accession numbers are ubiq7 [GenBank: DQ116441], *GhCPS1* [GenBank:574036.1]*, GhCPS2* [GenBank:574037.1] and *GhCPS3* [GenBank:574038.1]. Amplicon length is included in table under qPCR validation. *In silico* screen were performed with NCBI Blast and can be obtained from above web site. Primers were designed in exons of the gene. No splice variants were targeted.

| **qPCR OLIGONUCLEOTIDES** |
| --- |
| Primer sequences |
| RTPrimerDB Identification Number |
| Probe sequences |
| Location and identity of any modifications |
| Manufacturer of oligonucleotides |
| Purification method |

Primer sequences are included in the manuscript in the Material and Method section. No modifications were used. Primers were purchase from Integrated DNA Technologies and are salt-free.

| **qPCR PROTOCOL** |
| --- |
| Complete reaction conditions |
| Reaction volume and amount of cDNA/DNA |
| Primer, (probe), Mg++ and dNTP concentrations |
| Polymerase identity and concentration |
| Buffer/kit identity and manufacturer |
| Exact chemical constitution of the buffer |
| Additives (SYBR Green I, DMSO, etc.) |
| Manufacturer of plates/tubes and catalog number |
| Complete thermocycling parameters |
| Reaction setup (manual/robotic) |
| Manufacturer of qPCR instrument |

Each qPCR reaction had a 25 µl reaction volume containing:

cDNA corresponding to 25ng input RNA

1 µl 10 mM of each forward and reverse primer

12.5 µl 2 x iQ SYBR Green Super Mix (Bio-Rad, Cat#170-8880)

iQ 96-well PCR plates (Cat#223-9441) and plate sealers(Cat#MSB-1001) were purchased from BioRad.

Cycling parameters were:

Cycle 1: ( 1X)

Step 1: 95.0ºC for 03:00

Cycle 2: ( 40X)

Step 1: 95.0ºC for 00:10

Step 2: 60.0ºC for 00:45

Data collection and real-time analysis enabled.

Cycle 3: ( 1X)

Step 1: 95.0ºC for 01:00

Cycle 4: ( 1X)

Step 1: 60.0ºC for 01:00

Cycle 5: ( 80X)

Step 1: 60.0ºC for 00:10

Increase setpoint temperature after cycle 2 by 0.5ºC

Melt curve data collection and analysis enabled.

Reactions were set up manually in a designated room using designated equipment. qPCRs were performed with iCycler iQ^TM^ Real-Time PCR Dection System from Bio-Rad.

| **qPCR VALIDATION** |
| --- |
| Evidence of optimisation (from gradients) |
| Specificity (gel, sequence, melt, or digest) |
| For SYBR Green I, Cq of the NTC |
| Standard curves with slope and y-intercept |
| PCR efficiency calculated from slope |
| Confidence interval for PCR efficiency or standard error |
| r2 of standard curve |
| Linear dynamic range |
| Cq variation at lower limit |
| Confidence intervals throughout range |
| Evidence for limit of detection |
| If multiplex, efficiency and LOD of each assay. |

The specificity of the amplification products have been confirmed by size estimations on a 1.5% agarose gel and by analyzing their melting curves. Without a template, no Cq could be determined since it never passed the threshold line.

|  | **length (bp)** | **slope** | **y-intercept** | **% efficiency** | **r2** |
| --- | --- | --- | --- | --- | --- |
| **Ubiq7** | 198 | -3.448 | -2.196 | 93.5 | 0.990 |
| **GhCPS1** | 193 | -3.391 | -2.868 | 97.2 | 0.985 |
| **GhCPS2** | 230 | -3.507 | -0.602 | 92.8 | 0.999 |
| **GhCPS3** | 177 | -3.602 | -0.797 | 89.5 | 0.999 |

| **DATA ANALYSIS** |
| --- |
| qPCR analysis program (source, version) |
| Cq method determination |
| Outlier identification and disposition |
| Results of NTCs |
| Justification of number and choice of reference genes |
| Description of normalisation method |
| Number and concordance of biological replicates |
| Number and stage (RT or qPCR) of technical replicates |
| Repeatability (intra-assay variation) |
| Reproducibility (inter-assay variation, %CV) |
| Power analysis |
| Statistical methods for result significance |
| Software (source, version) |
| Cq or raw data submission using RDML |

qPCR analysis program (source, version): Bio-Rad, iCycler iQ Optical System Software (Version 3.1)

Cq’s were determined by the software automatically. By default, the baseline cycles and the threshold are automatically calculated. The automatic threshold calculation is done to use standards defined on the experimental plate, the threshold is adjusted to attain the highest possible correlation coefficient value for the standard curve.

Results of NTCs: no amplification products present thus no Cqs

Justification of number and choice of reference genes: cDNAs had previously been tested with

another reference gene (*Ubiq1*) with the similar results

Description of normalization method: endogenous reference gene

Number and concordance of biological replicates: 1

Number and stage (RT or qPCR) of technical replicates: 3 at qPCR level, 2 for RT Repeatability (intra-assay variation): was below one Cq
